# Correlates of cigarette smoking among male college students in Karachi, Pakistan

**DOI:** 10.1186/1471-2458-7-312

**Published:** 2007-11-01

**Authors:** Shafquat Rozi, Zahid A Butt, Saeed Akhtar

**Affiliations:** 1 Division of Epidemiology and Biostatistics, Department of Community Health Sciences, Aga Khan University, Stadium Road, PO Box 3500, Karachi, Pakistan; 2 World Health Organization, National Campaign Cell, PO Box 44000, NIH, Chak Shahzad, Islamabad, Pakistan; 3 Department of Community Medicine & Behavioral Sciences, Faculty of Medicine, Kuwait University, PO Box 24923, Safat 13110, Kuwait

## Abstract

**Background:**

About 1.3 billion people are regular smokers world wide and every day between 8,200 and 9,900 young people start to smoke, risking rapid addiction to nicotine. Transition from high school to college is a critical period to adopt healthy habits and life style. Therefore, it is important to understand the factors that might influence their smoking habit. Our study aims to assess the influence of factors that encourage college students to smoke cigarettes.

**Methods:**

The data used in this survey were obtained from a representative sample of registered colleges of Karachi. A random sample of 576 male college students of ages ranging from 15–30 years was interviewed using a questionnaire administered by survey officers, by applying multi stage cluster sampling during the academic year 2004–2005.

**Results:**

In this study, we found 26.7% of students had ever tried smoking, whereas 24%(95% CI: 21.0%–28.0%) of college students reported current smoking (that is whether one had smoked a cigarette in past 30 days). Among different age groups, prevalence of current smoking was 19.2% in 15–17 years, 26.5% in 18–20 years and 65% in 21 years and above. After adjusting for age of respondent, students in public schools were more likely to smoke as compared to students in private schools (adjusted OR = 2.3; 95% CI: 1.3–4.2). Students whose friends are smokers were 5 times more likely to smoke compared to those whose friends are non-smokers (adjusted OR = 4.8; 95%CI: 3.1 – 7.4). Those students having fathers with no formal schooling were more likely to smoke (adjusted OR = 2.2; 95% CI: 1.1–4.2) as compared to those whose fathers had some degree of education. Students having non-working mothers were more likely to smoke as compared to students with working mothers (adjusted OR = 2.8; 95% CI: 0.9–9.1). Students belonging to Bin Qasim (adjusted OR = 2.1; 95% C.I: 1.1–4.1) and Gadap town (adjusted OR = 2.1; 95%C.I) were more likely to smoke as compared to students residing in other towns.

**Conclusion:**

This study shows that smoking is strongly associated with age, which may suggest social tolerance to smoking in this setting and that social and educational variables appear to play a significant role in smoking among college students. Our study suggests that such factors should be taken into account when designing effective tobacco control programs among college students. This is an effort which has been done to reduce tobacco consumption among college students and introduce awareness programs to amend their health risk behavior.

## Background

Tobacco is a major global contributor to deaths from chronic diseases. There are about 1.3 billion smokers in the world and approximately 80% of them live in the developing countries [1]. Globally, there are 5 million deaths per year from tobacco use which are expected to rise to 10 million by the year 2025. It is also alarming to note that approximately 7 million of these will be from developing countries. Overall, the mortality and morbidity from tobacco use incurs an economic cost of US$ 200 billion annually [2]. According to World Health Organization (WHO) estimates, approximately 47% of men and 12% of women smoke worldwide. In developing countries, 48% of men and 7% of women smoke, while in developed countries, 42% of men smoke as compared to 24% of women [3]. The Global Youth Tobacco Survey (GYTS) conducted in 131 countries which surveyed 750,000 students of ages 13–15 years found that approximately 9% of students were current smokers while 11% currently used tobacco products other than cigarettes [4].

The National survey of US college students on tobacco use indicated that more than half (61%) of the students had used a tobacco product in the past while one-third currently used tobacco with cigarettes accounting for majority of tobacco use [5]. A survey of tobacco use by Massachusetts public college students showed a prevalence of current tobacco use in one third of respondents while nearly half of them (46.4%) had used tobacco in the past year [6]. Another study on US college students focusing on age of initiation found that 23% of students who had started smoking before the age of 10 were current smokers whereas 61% of those who began using smokeless tobacco before the age of 10 were current smokeless tobacco users [7]. Studies from developing countries have also reported a high prevalence of tobacco use among students. A survey of college students on tobacco use in Karnataka, India reported that 45% of students had used tobacco products [8]. A similar study conducted in Kerala, India on adolescents revealed prevalence; of current tobacco use of 11% and of current smoking to be 8% among boys aged 12–19 years [9]. Another study conducted in Karachi, Pakistan reported a prevalence of current smoking among adolescents to be 13.7% [10].

Several factors have been attributed to the use of tobacco products by students. These are the perceptions that smoking enhanced one's image, relieved boredom and helped in easing tension [8]. Another study in India identified use of tobacco by fathers and friends, older age, poor educational performance and availability to pocket money as major contributors to tobacco use by students [9]. In Pakistan, a survey of students' revealed similar factors like smoking by peers, family members and spending leisure time outside home as contributing to cigarette smoking [10].

Tobacco use and especially cigarette smoking is a major public health issue among students not only in developed countries but also among developing countries. The GYTS conducted in three cities of Pakistan focused on students in school who were 13 to 15 years of age [11-13]. Our survey is an attempt to study college going students as smoking is usually initiated during the period when adolescents are in college. Therefore, it should be a public health priority to educate this group regarding the hazards of smoking, so that their behavior can be modified. However, before initiating any awareness programs, it is important to understand the factors contributing to smoking among college students and design effective interventions to prevent it. This study was undertaken with the aim of estimating the prevalence of smoking among male college students and to identify the factors associated with smoking among these students in Karachi, Pakistan.

## Methods

A cross-sectional study was conducted from January through May 2005 at government and private colleges in Karachi. Presently, Karachi has been divided into 18 administrative towns [14]. For the purpose of this study, three towns were selected namely Gadap, Bin Qasim and Malir. There is an ethnic mix of people residing in these three towns. Socio-economically, the population belongs to the upper, middle and lower classes [14]. The population of these three towns in this survey was therefore considered to be representative of the population of Karachi.

Ethical approval for the study was obtained from the Aga Khan University's Ethical Review Committee (ERC). ERC is concerned with all research projects involving human subjects, whether as individuals or communities. In addition, we obtained a written permission from Nazim (administrative in charge of town) and district education officer to administer the study questionnaire in summer 2005 in college of their region. The research study received written consent from all selected colleges of the town prior to data collection. We agreed that the results will not be reported for individual institutions. Our study sample comprised of male college students. The rationale for surveying only male students was based on findings from GYTS conducted in three cities of Pakistan which showed a very low prevalence of current smoking among female students [11-13].

The selected students were initially explained the purpose of the study and also assured that the responses will not be disclosed to any college authority and privacy would be respected (Informed verbal consent). We used a multi-stage cluster sampling design based on college type. The first stage sampling frame contained 12 sampling units with stratification of colleges on their types (private and public). Depending on enrollment size, every xth student was selected from the students registering a random starting point. Of the students present on the day of our visit, 576 students were selected with a probability proportional to enrollment size of each college. The overall response rate to the study was 100%. Six trained interviewers (survey officers), age ranging from 20 to 30 years belonging to middle socioeconomic group and having at least a Bachelor's degree, who were fluent in English and their local language interviewed college students about smoking behavior, socio-demographic factors including age, ethnicity, religion, highest level of parental education, occupation of parents (proxy indicator for socio-economic status), smoking history of family/friend, number of sibling and place of residence. Smoking behavior of the student was assessed by asking whether the individual had smoked in his life or not, age and particular reason for initiation of smoking. A question about frequency of smoking was also asked to the students. The outcome variable smoking status (smoker or non-smoker) was assessed based on 30 days prevalence of cigarette smoking (that is whether one had smoked a cigarette in past 30 days) [15-18]. For smokers, current frequency of smoking was further categorized as daily, weekly, monthly and occasional smoker.

Descriptive statistics included mean (± standard deviation) for continuous and proportion for categorical variables were computed. To identify the factors associated with smoking among college students, associations between outcome variable (smoker & non-smoker) and independent variable were sought. Crude odds ratio (OR) and their 95% confidence interval (CI) were calculated by univariate logistic regression analysis. Variables with P ≤ 0.25 or biologically meaningful were selected for multivariate analysis [19]. To assess the independent effect of individual factors and control potential confounders, multiple logistic regression analysis was used and adjusted OR (AOR) with their 95% CIs were computed. After arrival of main effect model, plausible interactions were evaluated for inclusion in multivariate model. Statistical package Epi-Info version 6 [20] was used to enter the data and analysis was performed by using SPSS version 10 [21].

## Results

Socio-demographic characteristics are shown in Table 1. A total of 576 subjects were interviewed, 285 (49.5%) were from public and 291(50.5%) of them were from private colleges. There were five major ethnic groups Urdu (29.2%), Sindhi (24.1%), Punjabi (20.1%), Pushto (4.2%), Balochi (1.0%) and other ethnic groups comprised of Saraiki and Hindko (10.4%). Majority of students were residents of Bin-Qasim town (55.2%). About 27.2% of students stated that their parents are smoker, whereas (29.7%) stated that their uncle smoked and students also reported brother (6.9%), grandparents (6.1%) and cousins (6.1%) as smokers. Proportion of friends smoking (42.5%) was also noticeable. We found 26.7% of students who had ever tried smoking. Whereas in the study sample (139/576) or 24% (95% CI; 21%–28%) of college students reported current smoking while the prevalence among different age groups was 19.2% in 15–17 years, 26.5% in 18–20 years and 65% in 21 years and above. The median age of starting smoking was 16 years. However, age group 14–17 years was found to be more vulnerable, 63% (97/154) to acquire the habit of smoking. About 37% of college students stated advertisement and enjoyment as the reasons for starting smoking and 25.3% started due to peer pressure in spite of the fact that majority (87%) of students were aware of benefits of quitting smoking. There was significant difference in type of schools between smoker and non-smoker groups (Table 2). We also found that those students who reported friends smoking were more likely to be smoker than were those whose friends are non-smoker. Students who spend their leisure time in some activities and keep themselves busy in reading books, playing games, avail computer facilities were less likely to be smokers. Whereas, watching tobacco promotion ads on electronic media was significantly associated with respondent smoking status (p ≤ 0.25). The final multiple logistic regression model indicates that various socio-demographic characteristics are associated with smoking status of college students (Table 3). After adjusting for age of respondent, students in public schools were more likely to smoke as compared to students in private schools (adjusted OR = 2.3; 95% CI: 1.3–4.2). Students whose friends are smoker were 5 times more likely to smoke as compared to those whose friends are non-smoker (adjusted OR = 5.1; 95%CI: 3.2 – 7.9). Those students whose fathers had no formal schooling were more likely to smoke (adjusted OR = 2.2; 95% CI: 1.1–4.2) as compared to those who had some degree of education. Students whose mothers were not working were more likely to smoke as compared to students with working mothers (adjusted OR = 2.8; 95% CI: 0.9–9.1). Regarding area of residence, students belonging to Bin Qasim (adjusted OR = 2.1; 95% C.I: 1.1–4.1) and Gadap town (adjusted OR = 2.1; 95%C.I) were more likely to smoke as compared to students residing in other towns.

**Table 1:** Socio-demographic characteristics of respondent and their families

**Characteristics**	**Frequency**	**Percentage (%)**
**Type of college**		
Government	285	49.5
Private	291	50.5
		
**Age of the respondent**		
15–17	292	50.7
18–20	264	45.8
21 or above	20	3.5
Average age of the respondent(SD)	17.6(1.5)	
		
**Residential area of respondent**		
Gadap Town	63	10.9
Bin Qasim Town	318	55.2
Malir Town	79	13.7
other	116	20.1
		
**Father's education level**		
No formal schooling	96	16.7
Primary (1–5)	28	4.9
Middle (6–8)	42	7.3
Matric (9–10)	127	22.0
Intermediate & above (11 onwards)	283	49.1
		
**Mother's education level**		
No formal schooling	303	52.6
Primary (1–5)	53	9.2
Middle (6–8)	48	8.3
Matric (9–10)	80	13.9
Intermediate & above (11 onwards)	92	16.0
		
**Number of siblings**		
0–3	161	28.0
4–7	329	57.1
8 or above	86	14.9
Average no. of siblings (S.E)	4.93 (2.4)	
		
**Family history of smoking**		
Parent smokes	157	27.2
Brother smokes	40	6.9
Uncle smokes	171	29.7
Grandparents smoke	35	6.1
Cousin smoke	23	3.9
		
**Friends smoking**		
Yes	245	42.5
No	331	57.5
		
**Ever smoked**		
No	422	73.3
Yes	154	26.7
		
**Age of starting smoking(n = 154)**		
10–13	22	14.3
14–17	97	63.0
18 or above	35	22.7
		
**Median age of starting smoking & percentiles**	16.0	
	25	15
	50	16
	75	17
		
**Current frequency of smoking(n = 154)**		
Daily	37	24.0
Once a week	27	17.5
Once a month	20	13.0
Occasionally	55	35.7
Not at all	15	9.8
		
**Quantity daily smoked(n = 37)**		
< 1	11	29.7
1–3	10	27.0
4–6	11	29.7
5 or above	5	13.5

**Table 2:** Univariate analysis of factors associated with smoking among male college students in Karachi, Pakistan.

**Characteristics**	**Smoking status**		**Crude OR**	**95% C.I for OR**
	Non-smoker n = 437	Smoker n = 139		

**Type of school**				
Private	232 (53.1)	59 (42.4)	1.0	-
Government	205 (46.9)	80 (57.1)	1.5	(1.0–2.2)
**Age of the respondent:**				
Average age of respondent (S.E)	17.5 (1.3)	18.1 (1.8)	1.3	(1.1 – 1.5)
**Father's education level**				
No formal schooling	205	78	1.7	(0.9 – 3.2)
Primary (1–5)	97	30	1.4	(0.7 – 2.7)
Middle (6–8)	34	8	1.1	(0.4 – 2.7)
Matric (9–10)	22	6	1.2	(0.4 – 3.5)
Intermediate & above (11 onwards)	79	17	1.0	-
**Mother's occupation**				
Not working	416	135	1	-
Working	21	4	1.7	(0.5 – 5.0)
**Area of Residence**				
Gadap	47	16	1.02	(0.5 – 2.1)
Bin-Qasim	241	77	0.959	(0.5 – 1.5)
Malir	62	17	0.823	(0.4 – 1.6)
Other	87	29	1.0	-
**Uncle smoking**				
No	316(72.3)	89(64.0)	1.0	
Yes	121(27.7)	50(36.0)	1.5	(0.9–2.1)
**Grant parent smoking**				
No	414(94.7)	127(91.4)	1.0	-
Yes	23(5.3)	12(8.6)	1.7	(0.8–3.5)
**Friends smoking**				
No	293(67.0)	38(27.3)	1.0	
yes	144(33.0)	101(72.7)	5.4	(3.5–8.2)
**Read Books**				
Yes	275(62.9)	76(54.7)	1.0	
No	162(37.1)	63(45.3)	1.4	(0.9–2.1)
**Play Games**				
Yes	264(60.4)	73(52.5)	1.0	
No	173(39.6)	66(47.5)	1.4	(0.9–2.0)
**Access computer in leisure time**				
Yes	417(95.4)	124(89.2)	1.0	
No	20(4.6)	15(10.8)	2.5	(1.2–5.1)
**Watch tobacco promotion adds on electronic media**				
No	232(53.1)	54(38.8)	1.0	
Yes	205(46.9)	85(61.2)	1.8	(1.2–2.6)

**Table 3:** Multivariate analysis of factors associated with smoking among male college students in Karachi, Pakistan.

**Variable**	**Adjusted odds ratio**	**95% C.I**
**Type of College**		
Private	1.0	-
Public	2.3	(1.3 – 4.2)
**Age of the respondent Friends smoking**	1.2	(1.0 – 1.4)
No	1.0	-
Yes	5.1	(3.2 – 7.9)
**Access computer in leisure time**		
Yes	1.0	-
No	2.1	(1.0 – 4.7)
**Father's education level**		
No formal schooling	2.2	(1.1 – 4.2)
Primary (1–5)	1.5	(0.7 – 3.2)
Middle (6–8)	1.1	(0.4 – 3.0)
Matric (9–10)	1.5	(0.4 – 4.9)
Intermediate & above(11 onwards)	1.0	-
**Mother's occupation**		
Not working	2.8	(0.9 – 9.1)
Working	1.0	-
**Area of Residence**		
Gadap	2.1	(0.9 – 4.7)
Bin-Qasim	2.1	(1.1 – 4.1)
Malir	0.7	(0.3 – 1.6)
Other	1	-

## Discussion

### Main Findings

In our study, the prevalence of current smoking (30 days prevalence) among college students studying in private and public colleges in three towns of Karachi was 24% (95% C.I. : 21–28%). Similar current smoking estimates of 22.3% [22] and 28.5%23 were found among college students in U.S.A in the years 1993 and 1997. A survey conducted on Massachusetts public colleges reported a prevalence of current smoking to be 29% [6] while another study reported a prevalence of 26.8%. However, an earlier survey conducted in U.S.A showed a prevalence of 14% among college students [7]. A study conducted in Karachi on school going adolescents reported a prevalence of current smoking to be 13.7% [10]. Although the age group and sample is different it does indicate a rise in prevalence of current smoking from school going adolescents to college students.

We found several factors that were associated with smoking in our survey and that might play a role in favoring smoking among college students. Students in government (public) colleges were more likely to smoke as compared to their colleagues in private colleges which is similar to another study conducted on school going adolescents in Karachi [10]. A possible explanation could be more restrictions placed on students in private colleges or more opportunities for healthy recreational activities like sports as compared to government colleges. Friends' smoking was found to be significant indicating a link between peer pressure and the development of smoking habit [9,23,24]. This is similar to a study conducted on college students in India [8]. The median age of starting smoking among college students was 16 years. At this age, adolescents are influenced more by their friends and are generally less affected by the lifestyles of their parents. However, other studies have reported parental or relatives smoking as an influence on initiation of smoking [10,25]. A possible explanation could be that as an adolescent behaviour pattern, they are more likely to report smoking by friends as an influencing factor for smoking rather than their parents. Also at this age, adolescents want to be part of a group and try not to be left out which explains their propensity to continue smoking. This study showed that students belonging to Gadap and Bin-Qasim towns were more likely to be smokers as compared to those residing in other towns which is consistent with findings from another study conducted in Karachi [10]. This indicates that individuals in some localities are more prone to smoking. In addition, there might be more shops selling tobacco products in these localities while students might have easy access to tobacco products from shops. We also found that students whose fathers had no formal education were more likely to smoke as compared to students having educated fathers. Furthermore, our study showed that students who had non working mothers were also more prone to smoking. These findings indicate an association between low socio-economic status and smoking, as students belonging to families with less resources were more likely to be smokers. The lack of education among parents could also be one of the reasons as the parents might not be aware of the ill-effects of smoking; therefore, they are less likely to advise their children against smoking. Our study also indicated a link between cigarette smoking and the lack of time spent on activities like accessing computers in leisure time. However, this could also indicate that some of them might not own or have access to a computer, resulting in them going outside and spending time with their friends who smoke or because of their belonging to poor families being prone to activities like smoking. The association between smoking and low levels of reading or low access to computer may point to low socio economic status and/or education factors as correlates of smoking in college students

## Limitations

As our study was based on self-reported information on cigarette smoking, it is quite likely that there is a chance of under-reporting of smoking status among the respondents and reported smoking status of their relatives and friends. Our study also indicates that certain towns are more likely to have students as smokers as compared to other towns. This finding needs to be further analyzed by future studies. Another limitation of our study was that we could not attain biomedical validation of the current smoking status of the respondents.

## Conclusion

The prevalence of smoking among college students is a cause for concern, and trend indicates a higher preponderance among government college students compared to private college students. This study also reports that the initiation of smoking among students is significantly associated with the smoking habits of their peers. Another interesting fact was the association between low socioeconomic status of the parents and smoking among students. There is also a need felt to involve college students in healthy recreational activities as indicated in our study. Therefore, any intervention should include concerted efforts to involve students in healthy recreational activities, initiate tobacco control programs in colleges which have shown to have a beneficial effect as indicated by studies in U.S colleges [6] and to create awareness among general public about ill effects of smoking through print and electronic media. As our study focuses on college students, the findings would be especially helpful to initiate effective tobacco control programs in colleges and would also serve as a template to conduct further studies on this topic in Pakistan.

## Competing interests

The author(s) declare that they have no competing interests.

## Authors' contributions

SR conceived of the study, participated in its design and coordination and performed the statistical analysis. ZAB participated in the design and statistical analysis and drafted the manuscript. SA participated in the design and review of the study. All authors read and approved the final manuscript.

## Pre-publication history

The pre-publication history for this paper can be accessed here:


